# Comparison of overall survival of gastric neoplasms containing neuroendocrine carcinoma components with gastric adenocarcinoma: a propensity score matching study

**DOI:** 10.1186/s12885-020-07281-7

**Published:** 2020-08-18

**Authors:** Jiahui Chen, Anqiang Wang, Ke Ji, Zhaode Bu, Jiafu Ji

**Affiliations:** Department of Gastrointestinal Surgery, Key Laboratory of Carcinogenesis and Translational Research (Ministry of Education), Peking University Cancer Hospital & Institute, No. 52 Fucheng Road, Haidian District, Beijing, 100142 China

**Keywords:** Neuroendocrine carcinoma, Gastric adenocarcinoma, Overall survival

## Abstract

**Background:**

Gastric neoplasms containing neuroendocrine carcinoma (NEC) components are rare malignancies with highly aggressive behavior and a poor prognosis and include pure NEC and mixed tumors containing NEC components. We aimed to investigate whether there is a distinct difference in overall survival (OS) between gastric neoplasms containing NEC components and gastric adenocarcinoma.

**Methods:**

Surgically resected gastric neoplasms containing NEC components (*n* = 180) and gastric adenocarcinomas (*n* = 785) from January 2013 to December 2019 at Peking University Cancer Hospital were retrospectively analysed. Patients were categorized into a surgical group and a neoadjuvant group and adjusted using propensity score matching. In the two groups, gastric neoplasms containing NEC components were divided into pure NEC and mixed tumors with less than 30% (< 30% G-HMiNEN), between 30 and 70% (G-HMiNEN) and more than 70% (> 70% G-HMiNEN) neuroendocrine carcinoma components. OS was compared between these groups and the gastric adenocarcinoma group.

**Results:**

The OS of gastric neoplasms containing neuroendocrine NEC components was poorer than that of gastric adenocarcinomas in the surgical group, regardless of whether the percentage of neuroendocrine cancer components was less than 30%, between 30 and 70%, more than 70% or 100%. Cox multivariable regression analysis suggested that tumor category (neoplasms containing NEC components or gastric adenocarcinoma) was an independent risk factor for prognosis. Interestingly, among patients receiving neoadjuvant therapy, the difference was not significant.

**Conclusions:**

Gastric neoplasms containing any proportion of NEC components had poorer overall survival than gastric adenocarcinoma in patients treated with surgery directly, indicating that these neoplasms are more malignant than gastric adenocarcinoma. Among the patients receiving neoadjuvant therapy, the difference in overall survival was not significant, which was in sharp contrast with the results of the surgery group, suggesting that neoadjuvant therapy may have a good effect in the treatment of these neoplasms.

## Background

Gastric neoplasms containing neuroendocrine carcinoma (NEC) components are a heterogeneous subgroup of gastric cancer with highly aggressive behavior and poor prognosis and include pure NECs and mixed tumors containing NEC components. Every year there are approximately 1 million new cases of gastric cancer worldwide, and gastric neoplasms containing NEC components account for approximately 0.1–0.6% of these cases [1, 2]. Given the low incidence, there is little comprehensive basic and clinical research to systematically guide the treatment of these gastric neoplasms, making the prognosis of these tumors unsatisfactory [3–7].

According to the 2017 World Health Organization (WHO) digestive neuroendocrine tumor classification, neuroendocrine neoplasm (NEN) can be divided into three categories based on Ki-67 levels and mitotic counts (× 10 HPF): Grade 1 (G1, Ki67 ≤ 2%, mitoses< 2), Grade 2 (G2, 3% < Ki67 ≤ 20%, 2 ≤ mitoses≤20), Grade 3 (G3, Ki67 > 20%, mitoses> 20) [8]. Meanwhile, the American Joint Committee on Cancer (AJCC) defines highly differentiated NEN as a neuroendocrine tumor (NET) and the poorly differentiated NEN as a neuroendocrine carcinoma (NEC) based on the degree of tumor cell differentiation. Generally, G1, G2, and rare well-differentiated G3 NENs belong to the NETs, while poorly differentiated G3 NENs belong to NECs [8, 9]. Gastric mixed neuroendocrine-non-neuroendocrine neoplasm (G-MiNEN) is a special type of gastric NEN that is defined as containing more than 30% of both neuroendocrine and non-neuroendocrine components [8], accounting for approximately 7% of all G-NENs and 25% of gastric neuroendocrine carcinomas (G-NECs) [4–6]. For those mixed tumors with less than 30% or more than 70% neuroendocrine carcinoma components, there is no uniform definition. Considering the heterogeneity of MiNEN and the malignancy degree of the different components in the tumor, La Rosa et al. [10, 11] proposed dividing MiNEN into three categories: high-grade, intermediate-grade and low-grade. High-grade MiNEN consists of NEC and carcinoma/adenoma, intermediate-grade MiMEN consists of NET and carcinoma, and low-grade MiNEN consists of NET and adenoma. Therefore, in this study, gastric high-grade mixed neuroendocrine-non-neuroendocrine neoplasm (G-HMiNEN) was defined as gastric cancer containing more than 30% of both neuroendocrine carcinoma and adenocarcinoma components.

Generally, the prognosis of mixed tumors is largely determined by the most malignant component. Kim et al. [12] found that G-NEC has shorter progression-free survival (PFS) than gastric adenocarcinoma. Huang et al. [13] found that the prognosis of patients with more than 50% of neuroendocrine cancer components is significantly poorer than that of patients with less than 50% components. All of these studies provide evidence that tumors containing neuroendocrine cancer components may contribute to a worse prognosis. Therefore, we hypothesized that a mixed tumor containing neuroendocrine carcinoma components would have a worse prognosis than pure adenocarcinoma alone. We sought to find studies on the overall survival (OS) comparison between G-HMiNEN and gastric adenocarcinoma but failed. Thus, we think that a study of the comparison of the OS of G-HMiNEN and gastric adenocarcinoma will provide a valuable supplement to current research on G-HMiNEN. To overcome the bias caused by the differences between the covariates in the comparison, we used propensity score matching (PSM) to match important factors such as age, gender, tumor location, tumor size, pathological staging, and adjuvant chemotherapy between the two groups, making the research results more reliable.

## Methods

### Patient selection

We retrospectively collected patients diagnosed with gastric NENs and underwent radical resection at Peking University Cancer Hospital, Beijing, from January 2013 to December 2019. The inclusion criteria were as follows: (1) pathologically confirmed pure NEC or tumor containing NEC components; (2) no other tumors were diagnosed before the operation; (3) complete clinicopathological information and survival information that could be obtained through follow-up. Patients diagnosed with cM1 or cT4b before surgery or died from perioperative complications were excluded from the study. Patients with gastric adenocarcinomas undergoing radical surgery were randomly selected for PSM analyses.

### Follow-up

We followed the patients at least twice a year. Serum tumor markers test, gastroscope, and computed tomography (CT) scans were used to reexamine patients after surgery. Depending on the patients’ status, Magnetic resonance imaging (MRI) and Positron emission tomography & computed tomography (PET-CT) were also considered. For patients who cannot regularly visit our center for postoperative examination, we use telephone follow-up to obtain survival information.

### Diagnosis and classification

We re-evaluated the diagnosis and classification of G-HMiNEN. Mixed tumors with less than 30% or more than 70% neuroendocrine carcinoma components were also included in this study, which were defined as < 30% G-HMiNEN and > 70% G-HMiNEN, respectively. A tumor consisting of 100% NEC is defined as pure NEC. All neuroendocrine tumors were identified, diagnosed, and classified by two independent pathologists in accordance with the 2017 WHO classification of tumors [8]. Neuroendocrine components were identified by histological features and immunohistochemical specificity marks, such as synaptophysin (Syn), chromogranin A (CgA), and neuro cell adhesion molecule (CD56 or NCAM). The tumor staging described in the study was based on the AJCC 8th Edition TNM Staging Guidelines [9]. All possible disagreements were discussed in our study group.

### Definition of variables and groups

In this study, patients were divided into a surgical group and a neoadjuvant group based on whether they had received neoadjuvant therapy before surgery. Patients in the surgery group were assessed by the pTNM staging system, while patients in the neoadjuvant treatment group were assessed by the ypTNM staging system. OS refers to the time from surgery to the last follow-up, the time of death, or the end of follow-up (e.g., loss of follow-up or other cause of death).

### Propensity score matching

To accurately compare the prognosis of G-HMiNEN and gastric adenocarcinoma, we employed PSM to balance the differences between the two groups. PSM was performed through the Pamatching 3.04 plug-in in SPSS 22.0 software. Logistic regression models were used to estimate propensity scores based on gender, age, tumor location, tumor size, and pathological staging. Given a 0.2 caliper width, 1:2 nearest neighbor matching was performed. The chi-squared test and Mann-Whitney U test were used to further verify the matching results.

### Statistical analysis

All statistical analyses were performed using SPSS 22.0 statistical software (IBM, United States). The chi-squared test and Mann-Whitney U test were used for statistical analysis of categorical variables and continuous variables, respectively. Kaplan-Meier method was used for the comparison of OS. The log-rank test was used to compare survival rates. Multivariable Cox proportional hazards models were used to identify predictors of survival outcome. *P* < 0.05 was regarded as the threshold of significance.

## Results

### Patient selection and PSM results

**Fig. 1:**
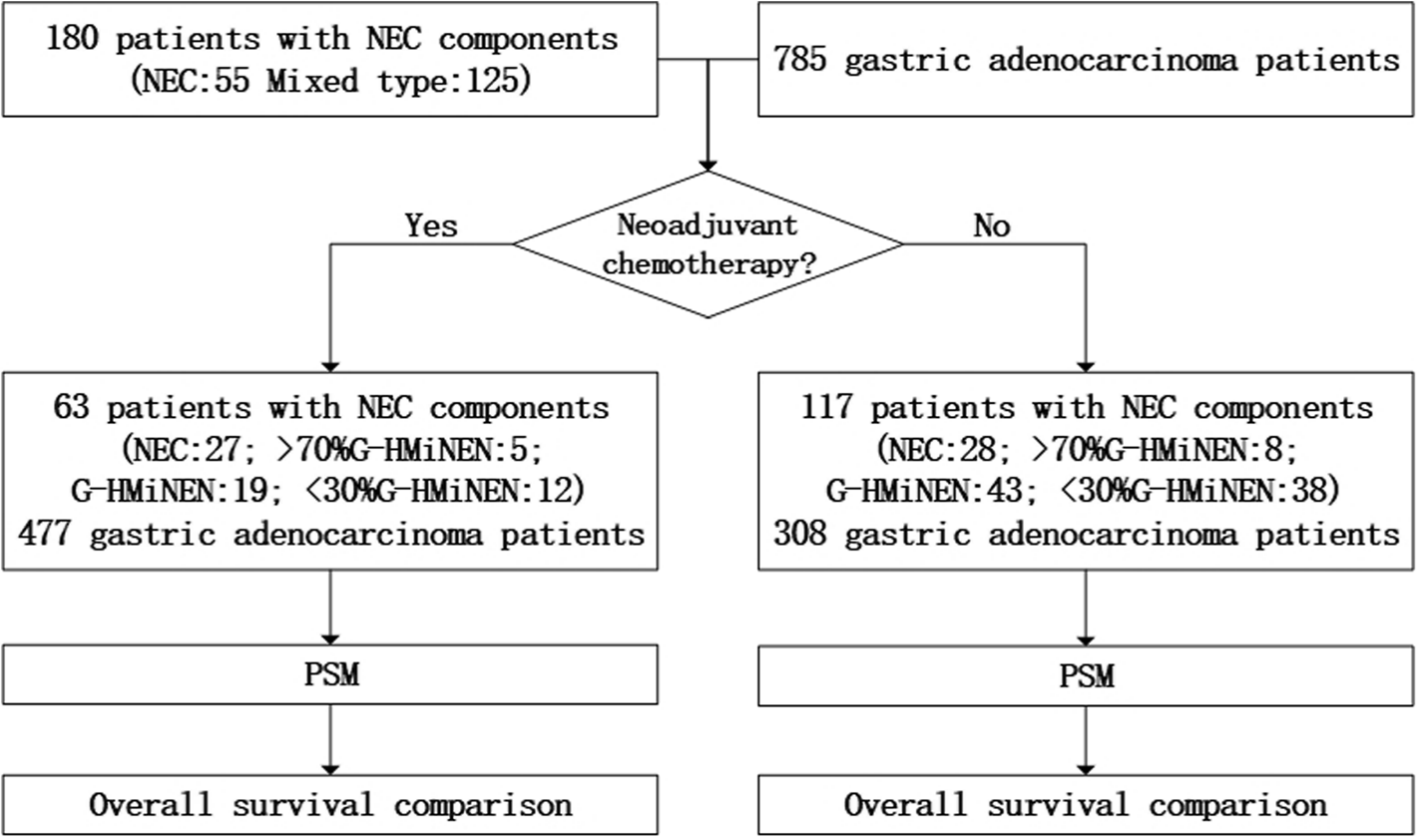
Flow chart of patient enrolment

Between 2013 and 2019, among the patients treated at the Gastrointestinal Cancer Center of Peking University Cancer Hospital, a total of 180 patients with gastric neoplasms containing NEC components met the inclusion criteria for the study, including 55 cases of pure NEC and 125 cases of mixed-type. Of these patients, a total of 65 patients received neoadjuvant therapy (NEC: 27, > 70% G-HMiNEN: 5, G-HMiNEN: 19, < 30% G-HMiNEN: 12), while the remaining 117 patients received surgery directly (NEC: 28, > 70% G-HMiNEN: 8, G-HMiNEN: 43, < 30% G-HMiNEN: 38). There were an insufficient number of patients in group > 70% G-HMiNEN group to conduct effective statistical analysis, so we combined the > 70% G-HMiNEN group with the NEC group for further analysis. We also randomly selected 785 patients with gastric adenocarcinoma who underwent radical surgery. Among them, 477 patients received neoadjuvant therapy, and the remaining 308 patients were treated with surgery directly (Fig. 1).

**Fig. 2:**
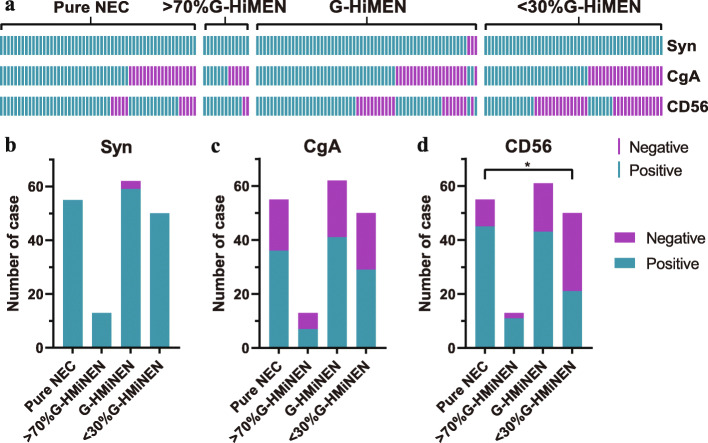
Illustrations of immunohistochemical staining patterns in gastric neoplasms containing NEC components. **a**. An overview of the expression of Syn, CgA, and CD56 in tumors containing NEC components. **b**. Syn expression in different NEC component groups. **c**. CgA expression in different NEC component groups. **d**. CD56 expression in different NEC component groups. CD56, neuro cell adhesion molecule; CgA, chromogranin A; NEC, neuroendocrine carcinoma; Syn, synaptophysin; *P*-value < 0.05 (*)

Immunohistochemical specificity markers were utilized to identify the neuroendocrine components (Fig. 2a). Syn was expressed in almost all neoplasms containing NEC components (98.3%), while the positive rates of CgA and CD56 were much lower (62.8 and 66.7%, respectively). No significant difference in the positive rate of Syn and CgA was observed between pure NEC, > 70% G-HMiNEN, G-HMiNEN, and < 30% G-HMiNEN (Fig. 2b, c), only the positive rate of CD56 was found to be higher in the pure NEC group than that in the < 30% G-HMiNEN group (Fig. 2d).

Therefore, prior to OS comparison, PSM was performed to ensure that there were no significant differences in patient gender, age, tumor location, tumor size, pathological staging, and adjuvant chemotherapy between the two groups.

### Comparison of OS between all patients with NEC components and patients with gastric adenocarcinoma in the surgical group and neoadjuvant group

**Fig. 3:**
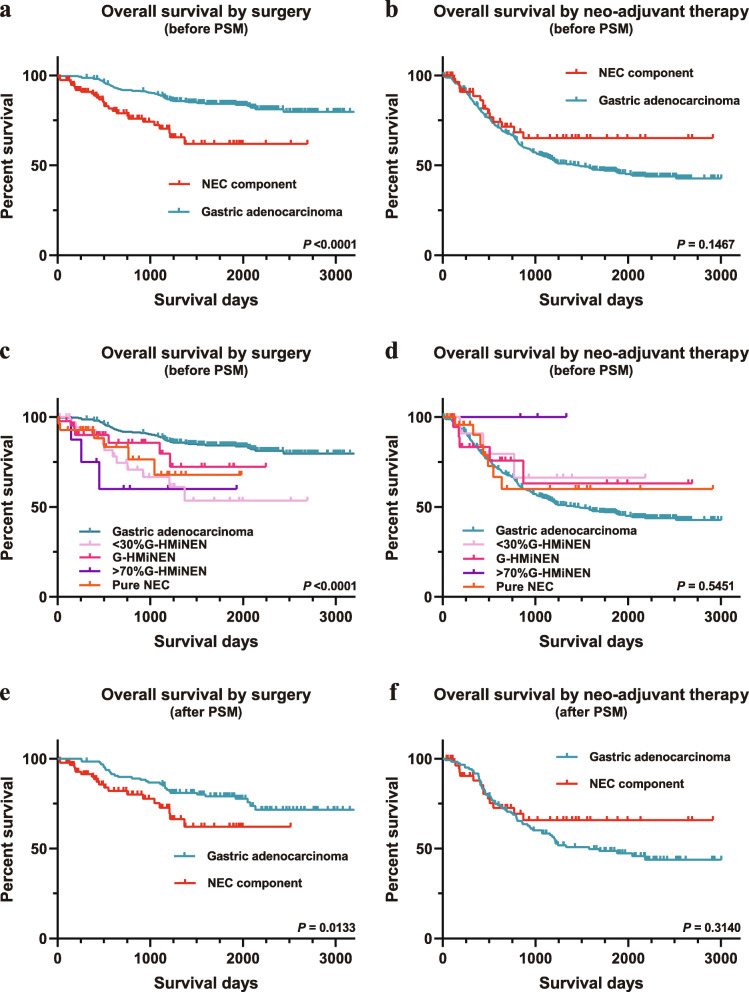
Comparison of OS between gastric neoplasms containing NEC components and gastric adenocarcinoma. **a**. OS comparison between gastric neoplasms containing NEC components and gastric adenocarcinoma before PSM in the surgical group. **b**. OS comparison between gastric neoplasms containing NEC components and gastric adenocarcinoma before PSM in the neoadjuvant group. **c**. OS comparison between different NEC content groups (pure NEC, > 70% G-HMiNEN, G-HMiNEN, and < 30% G-HMiNEN) and gastric adenocarcinoma before PSM in the surgical group. **d**. OS comparison between the different NEC content groups and gastric adenocarcinoma before PSM in the neoadjuvant group. **e**. OS comparison for patients in the surgical group after PSM. **f**. OS comparison for patients in the neoadjuvant group after PSM. NEC, neuroendocrine carcinoma; OS, overall survival; PSM, propensity score matching

Before PSM, we compared the survival curves between all patients with NEC components and patients with gastric adenocarcinoma by the Kaplan-Meier method (Fig. 3). Apparently, patients with NEC components had a poorer OS than those with gastric adenocarcinoma (Fig. 3a, *p* < 0.0001) in the surgical group. In contrast, no significant difference was observed between the patients receiving neoadjuvant therapy (Fig. 3b, *p* = 0.1467). According to the proportion of NEC components, patients were classified into pure NEC, > 70% G-HMiNEN, G-HMiNEN, and < 30% G-HMiNEN. The OS was also compared between patients with adenocarcinoma and these groups, and the results were similar to the overall comparison (Fig. 3c, d).

Before PSM, significant differences between the baseline characteristics were observed in the surgical group and the neoadjuvant group (Table 1 & Table 2). To balance the clinicopathological differences between the two groups, PSM was performed to ensure that there were no significant differences in patient gender, age, tumor location, tumor size, pathological staging, and adjuvant chemotherapy between the two groups. The detailed clinicopathological characteristics before and after PSM are shown in Table 1 and Table 2.

**Table 1:** Comparison of clinicopathological characteristics before and after PSM in surgical group

Patient Characteristics	Unmatched comparison			Matched comparison		
	Patients with NEC components (*n* = 117)	Gastric adenocarcinoma (*n* = 308)	*P* value	Patients with NEC components (*n* = 88)	Gastric adenocarcinoma (*n* = 128)	*P* value
Age (year), mean ± SD	61.7 ± 9.4	55.2 ± 10.8	< 0.001	59.7 ± 9.4	58.9 ± 9.7	0.551
Gender (male/female)	100/17	282/26	0.063	76/12	112/16	0.807
BMI, mean ± SD	24.1 ± 3.1	23.6 ± 3.6	0.100	24.4 ± 3.1	23.8 ± 3.6	0.182
Adjuvant therapy			< 0.001			0.189
Yes	87 (74.4)	109 (35.4)		58 (65.9)	73 (57.0)	
No	30 (25.6)	199 (64.6)		30 (34.1)	55 (43.0)	
Tumor location			< 0.001			0.679
Upper third	70 (59.8)	74 (24.0)		46 (52.3)	60 (46.9)	
Middle third	18 (15.4)	41 (13.3)		14 (15.9)	19 (14.8)	
Lower third	28 (23.9)	192 (62.3)		28 (31.8)	48 (37.5)	
Entire	1 (0.9)	1 (0.3)		0 (0.0)	1 (0.8)	
Tumor size			0.023			0.669
< 5 cm	80 (68.4)	243 (78.9)		63 (71.6)	95 (74.2)	
≥ 5 cm	37 (31.6)	65 (21.1)		25 (28.4)	33 (25.8)	
Type of gastrectomy			< 0.001			0.077
Total gastrectomy	78 (66.7)	79 (25.6)		53 (60.2)	59 (46.1)	
Distal gastrectomy	29 (24.8)	206 (66.9)		29 (33.0)	51 (39.8)	
Proximal gastrectomy	10 (8.5)	23 (7.5)		6 (6.8)	18 (14.1)	
Surgical procedure			< 0.001			0.001
Open	106 (90.6)	212 (68.6)		80 (90.9)	92 (71.9)	
Laparoscopic	11 (9.4)	96 (32.2)		8 (9.1)	36 (28.1)	
T stage			< 0.001			< 0.001
T1	16 (13.7)	195 (63.3)		14 (15.9)	53 (41.4)	
T2	26 (22.2)	18 (5.8)		25 (28.4)	12 (9.4)	
T3	48 (41.0)	49 (15.9)		27 (30.7)	35 (27.3)	
T4	27 (23.1)	46 (14.9)		22 (25.0)	28 (21.9)	
N stage			< 0.001			0.428
N0	48 (41.0)	210 (68.2)		37 (42.0)	62 (48.4)	
N1	32 (27.4)	26 (8.4)		22 (25.0)	22 (17.2)	
N2	20 (17.1)	27 (8.8)		16 (18.2)	16 (18.2)	
N3	17 (14.5)	45 (14.6)		13 (14.8)	24 (18.8)	
M stage			0.216			0.406
M0	117 (100.0)	304 (98.7)		88 (100.0)	127 (99.2)	
M1	0 (0.0)	4 (1.3)		0 (0.0)	1 (0.8)	
pTNM stage			< 0.001			0.399
I	30 (25.6)	202 (65.6)		30 (34.1)	56 (43.8)	
II	47 (40.2)	32 (10.4)		26 (29.5)	30 (23.4)	
III	40 (34.2)	70 (22.7)		32 (36.4)	41 (32.0)	
IV	0 (0.0)	4 (1.3)		0 (0.0)	1 (0.8)	

*BMI* Body Mass Index, *MiNEN* Mixed neuroendocrine-non-neuroendocrine neoplasm, *NEC* neuroendocrine carcinoma, *PSM* Propensity Score Matching

Patients with NEC components: NEC, < 30% high grade MiNEN, high grade MiNEN and > 70% high grade MiNEN

**Table 2:** Comparison of clinicopathological characteristics before and after PSM in neoadjuvant group

Patient Characteristics	Unmatched comparison			Matched comparison		
	Patients with NEC components (*n* = 63)	Gastric adenocarcinoma (*n* = 477)	*P* value	Patients with NEC components (*n* = 60)	Gastric adenocarcinoma (*n* = 120)	*P* value
Age (year), mean ± SD	61.2 ± 9.6	58.3 ± 10.3	0.037	61.3 ± 9.7	60.2 ± 9.5	0.484
Gender (male/female)	52/11	367/110	0.316	50/10	104/16	0.549
BMI, mean ± SD	24.1 ± 3.9	23.5 ± 3.4	0.285	23.9 ± 3.9	23.8 ± 3.4	0.855
Adjuvant therapy			0.124			0.173
Yes	58 (92.1)	459 (96.2)		56 (93.9)	117 (97.5)	
No	5 (7.9)	18 (3.8)		4 (6.7)	3 (2.5)	
Tumor location			< 0.001			0.361
Upper third	46 (73.0)	222 (46.5)		43 (71.7)	79 (65.8)	
Middle third	9 (14.3)	50 (10.5)		9 (15.0)	13 (10.8)	
Lower third	7 (11.1)	189 (39.6)		7 (11.7)	21 (17.5)	
Entire	1 (1.6)	16 (3.4)		1 (1.7)	7 (5.8)	
Tumor size			0.325			0.594
< 5 cm	36 (57.1)	303 (63.5)		33 (55.0)	71 (59.2)	
≥ 5 cm	27 (42.9)	174 (36.5)		27 (45.0)	49 (40.8)	
Type of gastrectomy			< 0.001			0.974
Total gastrectomy	50 (79.4)	239 (50.1)		47 (78.3)	94 (78.3)	
Distal gastrectomy	7 (11.1)	181 (37.9)		7 (11.7)	15 (12.5)	
Proximal gastrectomy	6 (9.5)	57 (11.9)		6 (10.0)	11 (9.2)	
Surgical procedure			0.394			1.000
Open	60 (95.2)	440 (92.2)		57 (95.0)	114 (95.0)	
Laparoscopic	3 (4.8)	37 (7.8)		3 (5.0)	6 (5.0)	
T stage			< 0.001			0.065
T0	1 (1.6)	0 (0.0)		0 (0.0)	0 (0.0)	
T1	3 (4.8)	28 (5.9)		3 (5.0)	3 (2.5)	
T2	3 (4.8)	60 (12.6)		3 (5.0)	15 (12.5)	
T3	35 (55.6)	154 (32.3)		34 (56.7)	47 (39.2)	
T4	21 (33.3)	235 (49.6)		20 (33.3)	55 (45.8)	
N stage			0.017			0.186
N0	17 (27.0)	158 (33.1)		16 (26.7)	44 (36.7)	
N1	22 (34.9)	99 (20.8)		21 (35.0)	33 (27.5)	
N2	14 (22.2)	80 (16.8)		14 (23.3)	17 (14.2)	
N3	10 (15.9)	140 (29.4)		9 (15.0)	26 (21.7)	
M stage			0.041			0.721
M0	60 (95.2)	471 (98.7)		59 (98.3)	117 (97.5)	
M1	3 (4.8)	6 (1.3)		1 (1.7)	3 (2.5)	
ypTNM stage			0.001			0.950
0	1 (1.6)	0 (0.0)		0 (0.0)	0 (0.0)	
I	4 (6.3)	52 (10.9)		4 (6.7)	6 (5.0)	
II	31 (49.2)	163 (34.2)		31 (51.7)	64 (53.3)	
III	24 (38.1)	256 (53.7)		24 (40.0)	47 (39.2)	
IV	3 (4.8)	6 (1.3)		1 (1.7)	3 (2.5)	

*BMI* Body Mass Index, *MiNEN* Mixed neuroendocrine-non-neuroendocrine neoplasm, *NEC* neuroendocrine carcinoma, *PSM* Propensity Score Matching

Patients with NEC components: NEC, < 30% high grade MiNEN, high grade MiNEN and > 70% high grade MiNEN

As a result, 88 patients with NEC components and 128 patients with gastric adenocarcinoma were matched in the surgical group (Table 1). Patients with NEC components also had a poorer OS than those with gastric adenocarcinoma (Fig. 3e, *p* = 0.0133). Multivariable analysis showed that adjuvant therapy, tumor category, and TNM stage were independent prognostic factors (Table 3).

**Table 3:** Univariate and multivariate analyses of survival after PSM in surgical group

Patient Characteristics	Univariate analysis			Multivariate analysis		
	HR	95% CI	*P* value	HR	95% CI	*P* value
Age (year)	0.993	0.966–1.021	0.608			
Gender						
male vs. female	0.542	0.271–1.084	0.083			
BMI	0.989	0.915–1.069	0.782			
Adjuvant therapy						
Yes vs. No	9.490	3.411–26.400	**< 0.001**	23.434	2.348–222.841	**0.007**
Tumor size						
≥ 5 cm vs. < 5 cm	2.557	1.458–4.485	**0.001**	1.318	0.710–2.448	0.381
Tumor category						
Carcinoma with NEC component vs. Gastric adenocarcinoma vs.	2.067	1.150–3.715	**0.015**	1.955	1.050–3.642	**0.035**
Type of gastrectomy			**0.021**			0.230
Total gastrectomy	1	1	–	1	1	–
Distal gastrectomy	0.382	0.193–0.758	0.006	0.545	0.256–1.160	0.115
Proximal gastrectomy	0.643	0.268–1.543	0.323	1.130	0.449–2.844	0.795
Surgical procedure			0.499			
Laparoscopic vs. Open	0.959	0.491–1.874	0.904			
TNM stage			**< 0.001**			**0.009**
I	1	1	–	1	1	
II	3.678	1.254–10.756	0.018	0.176	0.021–1.484	0.110
III	10.657	4.161–27.296	< 0.001	0.487	0.062–3.802	0.492
IV	30.036	3.443–262.046	0.002	2.477	0.139–44.100	0.537

To investigate whether neoadjuvant therapy had an effect on OS, 60 patients with NEC components and 120 patients with gastric adenocarcinoma were matched in the neoadjuvant group (Table 2). Interestingly, Kaplan-Meier analysis showed that among patients receiving neoadjuvant therapy, there was still no significant difference in OS between the two groups (Fig. 3f, *p* = 0.3140).

### Comparison of OS between patients with different proportions of NEC components and patients with gastric adenocarcinoma

**Fig. 4:**
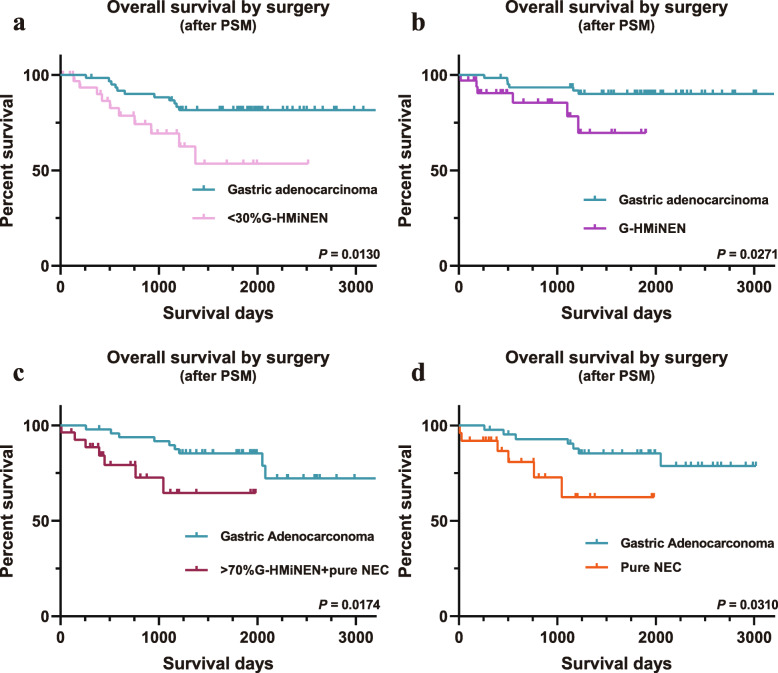
Comparison of OS between gastric neoplasm with different proportions of NEC and gastric adenocarcinoma in the surgical group. **a.** Overall survival comparison between < 30% G-HMiNEN and gastric adenocarcinoma. **b.** Overall survival comparison between G-HMiNEN and gastric adenocarcinoma. **c.** Overall survival comparison between > 70% G-HMiNEN plus pure NEC and gastric adenocarcinoma. **d.** Overall survival comparison between pure NEC alone and gastric adenocarcinoma

To investigate whether the level of NEC components had an effect on OS in the surgical group, < 30% G-HMiNEN, G-HMiNEN, pure NEC, and pure NEC plus > 70% G-HMiNEN were compared with gastric adenocarcinoma after PSM. The results showed that even the group with the lowest proportion of NEC components, the < 30% G-HMiNEN group, had a poorer OS than adenocarcinoma (Fig. 4a, *P* = 0.0130). As expected, the G-HMiNEN, pure NEC, and pure NEC plus > 70% G-HMiNEN groups, each with relatively high proportions of NEC components, had worse OS than the gastric adenocarcinoma group (Fig. 4b-d, *P* = 0.0271, 0.0174, 0.0310). Detailed clinical information after matching is shown in Additional file 1: Tables S1-S4.

**Fig. 5:**
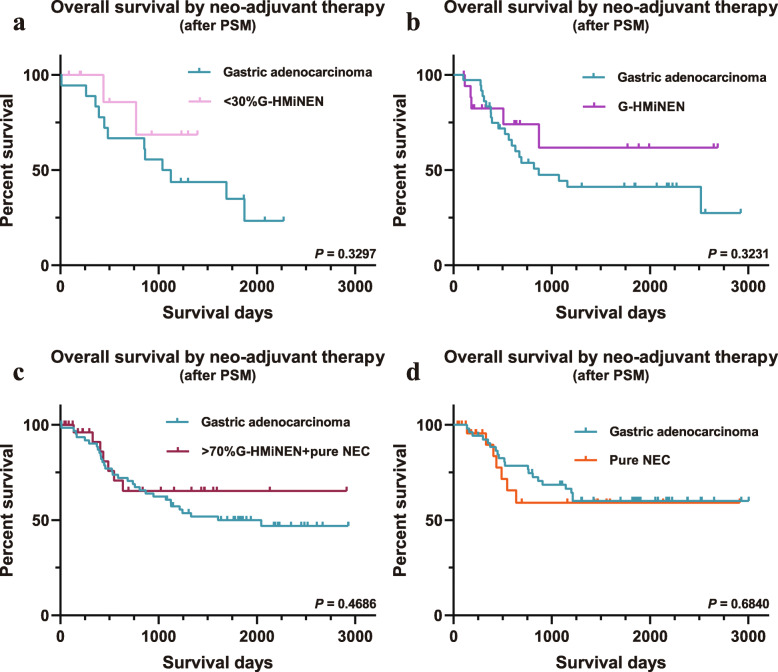
Comparison of OS between gastric neoplasm with different proportions of NEC components and gastric adenocarcinoma in the neoadjuvant group. **a**. Overall survival comparison between < 30% G-HMiNEN and gastric adenocarcinoma. **b**. Overall survival comparison between G-HMiNEN and gastric adenocarcinoma. **c**. Overall survival comparison between > 70% G-HMiNEN plus pure NEC and gastric adenocarcinoma. **d**. Overall survival comparison between pure NEC and gastric adenocarcinoma

PSM was also performed in the neoadjuvant group. In contrast to the results of the surgery group, in the pure NEC group (containing the highest proportion of NEC component), there was still no significant difference in OS from gastric adenocarcinoma (Fig. 5d). The other three groups with lower NEC content were also not significantly different from gastric adenocarcinoma in terms of OS (Fig. 5a-c). Detailed clinicopathological characteristics before and after PSM are shown in Additional file 1: Tables S5-S8.

## Discussion

Among gastric neuroendocrine neoplasms, the tumor containing NEC components is a special type, including pure NEC and mixed tumor containing NEC components. The incidence of these tumors is extremely low, but they are more invasive and have a poorer prognosis than well-differentiated G-NENs [4, 5].

In previous study, Kim et al. found that in patients who had not received neoadjuvant chemotherapy, progression-free survival (PFS) of pure G-NEC was poorer than that of gastric adenocarcinoma, while the PFS of mixed-type tumors was not significantly different from that of gastric adenocarcinoma [12]. In Kim’s study, the mixed type was defined as NET mixed with gastric cancer, rather than NEC. NET is much less malignant than NEC [14, 15]. This may be the reason why there was no significant difference in OS between mixed type and gastric adenocarcinomas. In addition, mixed tumors with less than 30% or more than 70% of NEC components were not included in that study, which we believe was a deficit of the study. PFS is an important indicator for evaluating prognosis, in many cases, it can reflect the trend of OS. Based on Kim’s research results, we regarded tumors containing NEC components as a whole and found that the OS of these tumors was poorer than that of adenocarcinoma in the surgical group. In the comparison of OS between mixed tumors with different proportions of NEC components and gastric adenocarcinoma, the results for pure NEC cases was similar to Kim’s. While the OS of mixed tumors was also poorer than that of gastric adenocarcinoma, whether the proportion of neuroendocrine cancer components was less than 30%, between 30 and 70%, or more than 70%, which was not mentioned in Kim’s study. Cox multivariable regression analysis showed that tumor category (neoplasm with NEC component or adenocarcinoma), tumor size, and TNM staging were independent risk factors for prognosis. This suggests that the prognosis of gastric neoplasms with NEC components is substantially different from that of gastric adenocarcinoma, and even a small percentage (< 30%) of NEC components can also impair prognosis, which challenges the current cut-off value of 30%.

The proportion of each component that must theoretically be greater than 30% was set in 1987 [16]. And since 2010, WHO has also adopted this standard to define MiNEN [16]. This largely avoids the overdiagnosis of MiNEN in tumors with only focal neuroendocrine marker expression and no corresponding morphological changes. In addition, it also prevents clinicians from dealing with these rare neoplasms too often without guidelines [15]. Nevertheless, it is now being questioned by an increasing number of scholars. The components in mixed tumors are not evenly distributed. For large tumors, the randomness of biopsy and postoperative pathological sampling causes the proportion of each component to fluctuate greatly, making it difficult to describe the proportion of each component precisely [15]. Park et al. compared the OS between tumors with more than 10% NEC components and gastric adenocarcinoma with or without less than 10% NEC, and they found that tumors with an NEC composition of more than 10% had a worse prognosis. This suggests that even a small proportion of malignant components can affect prognosis [3]. While in Park’s study, for unknown reasons, the authors did not compare the prognosis of mixed tumors with NEC components less than 10% with gastric adenocarcinomas directly, nor did they compare all NEC-containing tumors as a whole with gastric adenocarcinoma, which we believe was a deficit of the study. In our study, we regarded tumors containing NEC components as a whole and found that the OS of these tumors was poorer than that of adenocarcinoma in the surgical group. In addition, we also found that the OS of mixed tumors with less than 30%, between 30 and 70%, more than 70% NEC components or pure NEC was worse than that of gastric adenocarcinoma. Analysis of immunohistochemical markers show that there was no significant difference in the positive rate of Syn and CgA between different NEC content groups, only the positive rate of CD56 was found to be higher in the pure NEC group than that in the < 30% G-HMiNEN group. The role of CD56 in the diagnosis of NEC is still controversial. However, Syn and CgA are two well-recognized markers. Therefore, from the results of immunohistochemistry, we believed that there was no significantly difference in tumors containing NEC components. Studies on the molecular mechanism of pathogenesis show that NEC components and adenocarcinoma components have similar genomic abnormalities, similar losses of heterozygosity (LOH) and mutations at multiple loci and key oncogenes, such as *TP53*, *APC*, and *RB* genes. All these results imply that the two components in the mixed tumor may have a common origin and acquire biphenotypic differentiation during carcinogenesis [17–24]. Moreover, in the WHO definition of mixed neuroendocrine and non-neuroendocrine neoplasms of other organs (i.e., lung and thyroid) [25], no minimum percentage for either ingredient is established. Therefore, we believe that mixed tumors containing NEC components are actually of the same origin, have similar biological characteristics, and are different from gastric adenocarcinoma. We propose considering mixed tumors containing NEC components as a whole, rather than defining them based on the 30% definition for both tumor components, which has not been raised by other studies.

Previously, many studies have confirmed the efficacy of neoadjuvant chemotherapy in gastric adenocarcinoma [26, 27]. In a retrospective study involving 69 patients, Ma et al. found that neoadjuvant chemotherapy improves the survival of patients with NEC and HMiNEN of the stomach [28]. Van der Veen et al. reported that neoadjuvant chemotherapy could not benefit the survival of patients with mixed tumors containing NEC components [29]. However, because only eight patients were included in the neoadjuvant group, Van’s results are questionable. In our study, among patients receiving neoadjuvant therapy, no significant difference in OS between mixed tumor and gastric adenocarcinoma was observed. Even for the pure NEC group with the highest NEC content, there was no significant difference, suggesting that neoadjuvant therapy may have a positive effect on these neoplasms.

Although this is only a single-center retrospective study, the sample we reported is considerable for this rare disease, which can provide new ideas for clinical and basic research. In addition, we proposed treating all gastric neoplasms containing NEC components as a whole and found that neoadjuvant therapy may have a good effect on these neoplasms. In the future, we will conduct more genomics studies to confirm our ideas. This study also has its limitations. Due to the lack of recurrence and detailed chemotherapy information, we were unable to compare progression-free survival and analyse the effects of different chemotherapy regimens. As a retrospective study, despite our performing PSM in advance, selection bias cannot be completely avoided. In addition, since the exact proportion of each component in the mixed tumor could not be obtained, we could not determine whether there is a cutoff value for the diagnosis of the mixed tumor with NEC component less than 30%, so we could only treat all mixed tumors with NEC component as a whole.

## Conclusions

Our study demonstrated that gastric neoplasms with NEC components, regardless of the proportion of components, have poorer overall survival than gastric adenocarcinoma, indicating a higher degree of malignancy than gastric adenocarcinoma. Among the patients receiving neoadjuvant therapy, the difference in overall survival was not significant, which was in sharp contrast with the results of the surgery group, suggesting that neoadjuvant therapy may have a good effect on the prognosis of these malignancies. Therefore, for this type of malignancy, we should adopt more aggressive and powerful treatments than those used for gastric adenocarcinoma to improve the prognosis of patients. Neoadjuvant chemotherapy may be a good way to improve the efficacy of treatment for these tumors at advanced stages.

## Supplementary information

**Additional file 1: Table S1.** Comparison of clinicopathological characteristics before and after PSM of < 30%G-HMiNEN patients in surgical group. **Table S2.** Comparison of clinicopathological characteristics before and after PSM of G-HMiNEN patients in surgical group. **Table S3.** Comparison of clinicopathological characteristics before and after PSM of > 70%G-HMiNEN plus pure NEC patients in surgical group. **Table S4.** Comparison of clinicopathological characteristics before and after PSM of pure NEC patients in surgical group. **Table S5.** Comparison of clinicopathological characteristics before and after PSM of < 30%G-HMiNEN patients in neoadjuvant group. **Table S6.** Comparison of clinicopathological characteristics before and after PSM of G-HMiNEN patients in neoadjuvant group. **Table S7.** Comparison of clinicopathological characteristics before and after PSM of > 70%G-HMiNEN plus pure NEC patients in neoadjuvant group. **Table S8.** Comparison of clinicopathological characteristics before and after PSM of pure NEC patients in neoadjuvant group.

## Data Availability

The datasets used and/or analysed during the current study are available from the corresponding author on reasonable request.

## Abbreviations

AJCC: American Joint Committee on cancer
CT: Computed tomography
G-HMiNEN: Gastric high-grade mixed neuroendocrine-non-neuroendocrine neoplasm
G-NEC: Gastric neuroendocrine carcinoma
HPF: High power field
MiNEN: Mixed neuroendocrine-non-neuroendocrine neoplasm
NEC: Neuroendocrine carcinoma
NEN: Neuroendocrine neoplasm
NET: Neuroendocrine tumor
MRI: Magnetic resonance imaging
OS: Overall survival
PET-CT: Positron emission tomography & computed tomography
PFS: Progression-free survival
PSM: Propensity score matching
WHO: World Health Organization
