# Supplementary material for: Comparison of overall survival of gastric neoplasms containing neuroendocrine carcinoma components with gastric adenocarcinoma: a propensity score matching study
Source: BMC Cancer. 2020 Aug 18;20:777. doi: 10.1186/s12885-020-07281-7 (PMC7437076; doi:10.1186/s12885-020-07281-7)
Supplement: Supplementary file 1 — Additional file 1: Table S1. Comparison of clinicopathological characteristics before and after PSM of < 30%G-HMiNEN patients in surgical group. Table S2. Comparison of clinicopathological characteristics before and after PSM of G-HMiNEN patients in surgical group. Table S3. Comparison of clinicopathological characteristics before and after PSM of > 70%G-HMiNEN plus pure NEC patients in surgical group. Table S4. Comparison of clinicopathological characteristics before and after PSM of pure NEC patients in surgical group. Table S5. Comparison of clinicopathological characteristics before and after PSM of < 30%G-HMiNEN patients in neoadjuvant group. Table S6. Comparison of clinicopathological characteristics before and after PSM of G-HMiNEN patients in neoadjuvant group. Table S7. Comparison of clinicopathological characteristics before and after PSM of > 70%G-HMiNEN plus pure NEC patients in neoadjuvant group. Table S8. Comparison of clinicopathological characteristics before and after PSM of pure NEC patients in neoadjuvant group. [file 12885_2020_7281_MOESM1_ESM.docx]

**Supplement Table 1** Comparison of clinicopathological characteristics before and after PSM of <30%G-HMiNEN patients in surgical group

| Patient Characteristics | Unmatched comparison | | |  | Matched comparison | | |  |
| --- | --- | --- | --- | --- | --- | --- | --- | --- |
|  | <30%G-HMiNEN (n=38) | Gastric adenocarcinoma  (n=308) | *P* value |  | <30%G-HMiNEN (n=34) | Gastric adenocarcinoma  (n=61) | *P* value |  |
| Age (year), mean±SD | 59.9±11.1 | 55.2±10.8 | 0.012 |  | 59.3±11.4 | 59.2±10.5 | 0.977 |  |
| Gender (male/female) | 29/9 | 282/26 | 0.003 |  | 27/7 | 52/9 | 0.466 |  |
| BMI, mean±SD | 23.9±2.9 | 23.6±3.6 | 0.543 |  | 23.7±2.8 | 23.7±3.5 | 0.984 |  |
| Adjuvant therapy |  |  | <0.001 |  |  |  | 0.315 |  |
| Yes | 26 (68.4) | 109 (35.4) |  |  | 22 (64.7) | 33 (54.1) |  |  |
| No | 12 (31.6) | 199 (64.6) |  |  | 12 (35.5) | 28 (45.9) |  |  |
| Tumor location |  |  | <0.001 |  |  |  | 0.902 |  |
| Upper third | 22 (57.9) | 74 (24.0) |  |  | 19 (55.9) | 34 (55.7) |  |  |
| Middle third | 5 (13.2) | 41 (13.3) |  |  | 5 (14.7) | 9 (14.8) |  |  |
| Lower third | 10 (26.3) | 192 (62.3) |  |  | 10 (29.4) | 17 (27.9) |  |  |
| Entire | 1 (2.6) | 1 (0.3) |  |  | 0 (0.0) | 1 (1.6) |  |  |
| Tumor size |  |  | 0.271 |  |  |  | 0.980 |  |
| < 5cm | 27 (71.1) | 243 (78.9) |  |  | 25 (73.5) | 45 (73.8) |  |  |
| ≥5cm | 11 (28.9) | 65 (21.1) |  |  | 9 (26.5) | 16 (26.2) |  |  |
| Type of gastrectomy |  |  | <0.001 |  |  |  | 0.126 |  |
| Total gastrectomy | 26 (68.4) | 79 (25.6) |  |  | 23 (67.6) | 29 (47.5) |  |  |
| Distal gastrectomy | 9 (23.7) | 206 (66.9) |  |  | 9 (26.5) | 22 (36.1) |  |  |
| Proximal gastrectomy | 3 (7.9) | 23 (7.5) |  |  | 2 (5.9) | 10 (16.4) |  |  |
| Surgical procedure |  |  | 0.003 |  |  |  | 0.029 |  |
| Open | 35 (92.1) | 212 (68.6) |  |  | 31 (91.2) | 44 (72.1) |  |  |
| Laparoscopic | 3 (7.9) | 96 (32.2) |  |  | 3 (8.8) | 17 (27.9) |  |  |
| T stage |  |  | <0.001 |  |  |  | 0.008 |  |
| T1 | 7 (18.4) | 195 (63.3) |  |  | 6 (17.6) | 28 (45.9) |  |  |
| T2 | 9 (23.7) | 18 (5.8) |  |  | 9 (26.5) | 4 (6.6) |  |  |
| T3 | 11 (28.9) | 49 (15.9) |  |  | 9 (26.5) | 16 (26.2) |  |  |
| T4 | 11 (28.9) | 46 (14.9) |  |  | 10 (29.4) | 13 (21.3) |  |  |
| N stage |  |  | 0.038 |  |  |  | 0.661 |  |
| N0 | 18 (47.4) | 210 (68.2) |  |  | 16 (47.1) | 29 (47.5) |  |  |
| N1 | 5 (13.2) | 26 (8.4) |  |  | 4 (11.8) | 12 (19.7) |  |  |
| N2 | 8 (21.1) | 27 (8.8) |  |  | 7 (20.6) | 8 (13.1) |  |  |
| N3 | 7 (18.4) | 45 (14.6) |  |  | 7 (20.6) | 12 (19.7) |  |  |
| M stage |  |  | 0.480 |  |  |  |  |  |
| M0 | 38 (100.0) | 304 (98.7) |  |  | 34 (100.0) | 61 (100.0) |  |  |
| M1 | 0 (0.0) | 4 (1.3) |  |  | 0 (0.0) | 0 (9.9) |  |  |
| pTNM stage |  |  | <0.001 |  |  |  | 0.537 |  |
| I | 12 (31.6) | 202 (65.6) |  |  | 12 (35.3) | 28 (45.9) |  |  |
| II | 9 (23.7) | 32 (10.4) |  |  | 6 (17.6) | 11 (18.0) |  |  |
| III | 17 (44.7) | 70 (22.7) |  |  | 16 (47.1) | 22 (36.1) |  |  |
| IV | 0 (0.0) | 4 (1.3) |  |  | 0 (0.0) | 0 (0.0) |  |  |
| **BMI**: Body Mass Index; **PSM**: Propensity Score Matching; **<30%G-HMiNEN**: Gastric high-grade mixed neuroendocrine-non-neuroendocrine neoplasm with neuroendocrine carcinoma components less than 30% | | | | | | | | |

**Supplement Table 2** Comparison of clinicopathological characteristics before and after PSM of G-HMiNEN patients in surgical group

| Patient Characteristics | Unmatched comparison | | |  | Matched comparison | | |  |
| --- | --- | --- | --- | --- | --- | --- | --- | --- |
|  | G-HMiNEN  (n=43) | Gastric adenocarcinoma  (n=308) | *P* value |  | G-HMiNEN  (n=34) | Gastric adenocarcinoma  (n=62) | *P* value |  |
| Age (year), mean±SD | 61.9±9.3 | 55.2±10.8 | <0.001 |  | 60.2±8.9 | 59.0±8.3 | 0.520 |  |
| Gender (male/female) | 37/6 | 282/26 | 0.239 |  | 29/5 | 56/6 | 0.459 |  |
| BMI, mean±SD | 24.5±3.4 | 23.6±3.6 | 0.115 |  | 24.8±3.0 | 23.6±3.5 | 0.088 |  |
| Adjuvant therapy |  |  | <0.001 |  |  |  | 0.288 |  |
| Yes | 33 (76.7) | 109 (35.4) |  |  | 24 (70.6) | 37 (59.7) |  |  |
| No | 10 (23.3) | 199 (64.6) |  |  | 10 (29.4) | 25 (40.3) |  |  |
| Tumor location |  |  | <0.001 |  |  |  | 0.179 |  |
| Upper third | 27 (62.8) | 74 (24.0) |  |  | 20 (58.8) | 26 (41.9) |  |  |
| Middle third | 8 (18.6) | 41 (13.3) |  |  | 6 (17.6) | 10 (16.1) |  |  |
| Lower third | 8 (18.6) | 192 (62.3) |  |  | 8 (23.5) | 26 (41.9) |  |  |
| Entire | 0 (0.0) | 1 (0.3) |  |  | 0 (0.0) | 0 (0.0) |  |  |
| Tumor size |  |  | 0.019 |  |  |  | 0.180 |  |
| < 5cm | 27 (62.8) | 243 (78.9) |  |  | 22 (64.7) | 48 (77.4) |  |  |
| ≥5cm | 16 (37.2) | 65 (21.1) |  |  | 12 (35.3) | 14 (22.6) |  |  |
| Type of gastrectomy |  |  | <0.001 |  |  |  | 0.641 |  |
| Total gastrectomy | 29 (67.4) | 79 (25.6) |  |  | 21 (61.8) | 34 (54.8) |  |  |
| Distal gastrectomy | 10 (23.3) | 206 (66.9) |  |  | 10 (29.4) | 24 (38.7) |  |  |
| Proximal gastrectomy | 4 (9.3) | 23 (7.5) |  |  | 3 (8.8) | 4 (6.5) |  |  |
| Surgical procedure |  |  | 0.008 |  |  |  | 0.007 |  |
| Open | 38 (88.4) | 212 (68.6) |  |  | 31 (91.2) | 40 (64.5) |  |  |
| Laparoscopic | 5 (11.6) | 96 (32.2) |  |  | 3 (8.8) | 22 (35.5) |  |  |
| T stage |  |  | <0.001 |  |  |  | 0.035 |  |
| T1 | 6 (14.0) | 195 (63.3) |  |  | 6 (17.6) | 25 (40.3) |  |  |
| T2 | 7 (16.3) | 18 (5.8) |  |  | 6 (17.6) | 5 (8.1) |  |  |
| T3 | 25 (58.1) | 49 (15.9) |  |  | 17 (50.0) | 18 (29.0) |  |  |
| T4 | 5 (11.6) | 46 (14.9) |  |  | 5 (14.7) | 14 (22.6) |  |  |
| N stage |  |  | <0.001 |  |  |  | 0.608 |  |
| N0 | 16 (37.2) | 210 (68.2) |  |  | 13 (38.2) | 31 (50.0) |  |  |
| N1 | 15 (34.9) | 26 (8.4) |  |  | 9 (26.5) | 14 (22.6) |  |  |
| N2 | 4 (9.3) | 27 (8.8) |  |  | 4 (11.8) | 8 (12.9) |  |  |
| N3 | 8 (18.6) | 45 (14.6) |  |  | 8 (23.5) | 9 (14.5) |  |  |
| M stage |  |  | 0.452 |  |  |  | 1.000 |  |
| M0 | 43 (100.0) | 304 (98.7) |  |  | 34 (100.0) | 62 (100.0) |  |  |
| M1 | 0 (0.0) | 4 (1.3) |  |  | 0 (0.0) | 0 (0.0) |  |  |
| pTNM stage |  |  | <0.001 |  |  |  | 0.521 |  |
| I | 10 (23.3) | 202 (65.6) |  |  | 10 (29.4) | 25 (40.3) |  |  |
| II | 54 (51.2) | 32 (10.4) |  |  | 13 (38.2) | 22 (35.5) |  |  |
| III | 11 (25.6) | 70 (22.7) |  |  | 11 (32.4) | 15 (24.2) |  |  |
| IV | 0 (0.0) | 4 (1.3) |  |  | 0 (0.0) | 0 (0.0) |  |  |
| **BMI**: Body Mass Index; **PSM**: Propensity Score Matching; **G-HMiNEN**: Gastric high-grade mixed neuroendocrine-non-neuroendocrine neoplasm | | | | | | | | |

**Supplement Table 3** Comparison of clinicopathological characteristics before and after PSM of >70%G-HMiNEN plus pure NEC patients in surgical group

| Patient Characteristics | Unmatched comparison | | |  | Matched comparison | | |  |
| --- | --- | --- | --- | --- | --- | --- | --- | --- |
|  | >70%G-HMiNEN plus pure NEC (n=36) | Gastric adenocarcinoma  (n=308) | *P* value |  | >70%G-HMiNEN plus pure NEC  (n=27) | Gastric adenocarcinoma  (n=49) | *P* value |  |
| Age (year), mean±SD | 63.3±7.6 | 55.2±10.8 | <0.001 |  | 61.4±7.6 | 61.0±9.0 | 0.884 |  |
| Gender (male/female) | 34/2 | 282/26 | 0.549 |  | 25/2 | 46/3 | 0.829 |  |
| BMI, mean±SD | 24.0±3.0 | 23.6±3.6 | 0.452 |  | 24.5±3.0 | 23.0±3.3 | 0.044 |  |
| Adjuvant therapy |  |  | <0.001 |  |  |  | 0.653 |  |
| Yes | 28 (77.8) | 109 (35.4) |  |  | 19 (70.4) | 32 (65.3) |  |  |
| No | 8 (22.2) | 199 (64.6) |  |  | 8 (29.6) | 17 (34.7) |  |  |
| Tumor location |  |  | <0.001 |  |  |  | 0.501 |  |
| Upper third | 21 (58.3) | 74 (24.0) |  |  | 13 (48.1) | 18 (36.7) |  |  |
| Middle third | 5 (13.9) | 41 (13.3) |  |  | 4 (14.8) | 6 (12.2) |  |  |
| Lower third | 10 (27.8) | 192 (62.3) |  |  | 10 (37.0) | 25 (51.0) |  |  |
| Entire | 0 (0.0) | 1 (0.3) |  |  | 0 (0.0) | 0 (0.0) |  |  |
| Tumor size |  |  | 0.359 |  |  |  | 0.434 |  |
| < 5cm | 26 (72.2) | 243 (78.9) |  |  | 21 (77.8) | 34 (69.4) |  |  |
| ≥5cm | 10 (27.8) | 65 (21.1) |  |  | 6 (22.2) | 15 (30.6) |  |  |
| Type of gastrectomy |  |  | <0.001 |  |  |  | 0.776 |  |
| Total gastrectomy | 23 (63.9) | 79 (25.6) |  |  | 14 (51.9) | 23 (46.9) |  |  |
| Distal gastrectomy | 10 (27.8) | 206 (66.9) |  |  | 10 (37.0) | 22 (44.9) |  |  |
| Proximal gastrectomy | 3 (8.3) | 23 (7.5) |  |  | 3 (11.1) | 4 (8.2) |  |  |
| Surgical procedure |  |  | 0.004 |  |  |  | 0.011 |  |
| Open | 33 (91.7) | 212 (68.6) |  |  | 24 (88.9) | 30 (61.2) |  |  |
| Laparoscopic | 3 (8.3) | 96 (32.2) |  |  | 3 (11.1) | 19 (38.8) |  |  |
| T stage |  |  | <0.001 |  |  |  | 0.002 |  |
| T1 | 3 (8.3) | 195 (63.3) |  |  | 3 (11.1) | 17 (34.7) |  |  |
| T2 | 10 (27.8) | 18 (5.8) |  |  | 10 (37.0) | 3 (6.7) |  |  |
| T3 | 12 (33.3) | 49 (15.9) |  |  | 5 (18.5) | 16 (32.7) |  |  |
| T4 | 11 (30.6) | 46 (14.9) |  |  | 9 (33.3) | 13 (26.5) |  |  |
| N stage |  |  | <0.001 |  |  |  | 0.199 |  |
| N0 | 14 (38.9) | 210 (68.2) |  |  | 10 (37.0) | 21 (42.9) |  |  |
| N1 | 12 (33.3) | 26 (8.4) |  |  | 10 (37.0) | 11 (22.4) |  |  |
| N2 | 8 (22.2) | 27 (8.8) |  |  | 6 (22.2) | 8 (16.3) |  |  |
| N3 | 2 (5.6) | 45 (14.6) |  |  | 1 (3.7) | 9 (18.4) |  |  |
| M stage |  |  | 0.492 |  |  |  | 1.000 |  |
| M0 | 36 (100.0) | 304 (98.7) |  |  | 27 (100.0) | 49 (100.0) |  |  |
| M1 | 0 (0.0) | 4 (1.3) |  |  | 0 (0.0) | 0 (0.0) |  |  |
| pTNM stage |  |  | <0.001 |  |  |  | 0.834 |  |
| I | 8 (22.2) | 202 (65.6) |  |  | 8 (29.6) | 17 (34.7) |  |  |
| II | 16 (44.4) | 32 (10.4) |  |  | 10 (37.0) | 15 (30.6) |  |  |
| III | 12 (33.3) | 70 (22.7) |  |  | 9 (33.3) | 17 (34.7) |  |  |
| IV | 0 (0.0) | 4 (1.3) |  |  | 0 (0.0) | 0 (0.0) |  |  |
| **BMI**: Body Mass Index; **NEC**: neuroendocrine carcinoma; **PSM**: Propensity Score Matching; **>70%G-HMiNEN**: Gastric high-grade mixed neuroendocrine-non-neuroendocrine neoplasm with neuroendocrine carcinoma components over 70% | | | | | | | | |

**Supplement Table 4** Comparison of clinicopathological characteristics before and after PSM of pure NEC patients in surgical group

| Patient Characteristics | Unmatched comparison | | |  | Matched comparison | | |  |
| --- | --- | --- | --- | --- | --- | --- | --- | --- |
|  | pure NEC  (n=28) | Gastric adenocarcinoma  (n=308) | *P* value |  | pure NEC  (n=25) | Gastric adenocarcinoma  (n=43) | *P* value |  |
| Age (year), mean±SD | 62.9±7.5 | 55.2±10.8 | <0.001 |  | 62.5±7.8 | 60.7±8.0 | 0.387 |  |
| Gender (male/female) | 26/2 | 282/26 | 0.812 |  | 23/2 | 39/4 | 0.855 |  |
| BMI, mean±SD | 24.5±2.8 | 23.6±3.6 | 0.191 |  | 24.5±2.8 | 23.3±3.7 | 0.115 |  |
| Adjuvant therapy |  |  | <0.001 |  |  |  | 0.725 |  |
| Yes | 22 (78.6) | 109 (35.4) |  |  | 19 (76.0) | 31 (72.1) |  |  |
| No | 6 (21.4) | 199 (64.6) |  |  | 6 (24.0) | 12 (27.9) |  |  |
| Tumor location |  |  | 0.001 |  |  |  | 0.071 |  |
| Upper third | 17 (60.7) | 74 (24.0) |  |  | 14 (56.0) | 12 (27.9) |  |  |
| Middle third | 2 (7.1) | 41 (13.3) |  |  | 2 (8.0) | 5 (11.6) |  |  |
| Lower third | 9 (32.1) | 192 (62.3) |  |  | 9 (36.0) | 26 (60.5) |  |  |
| Entire | 0 (0.0) | 1 (0.3) |  |  | 0 (0.0) | 0 (0.0) |  |  |
| Tumor size |  |  | 0.393 |  |  |  | 0.631 |  |
| < 5cm | 24 (85.7) | 243 (78.9) |  |  | 22 (88.0) | 36 (83.7) |  |  |
| ≥5cm | 4 (14.3) | 65 (21.1) |  |  | 3 (12.0) | 7 (16.3) |  |  |
| Type of gastrectomy |  |  | <0.001 |  |  |  | 0.090 |  |
| Total gastrectomy | 17 (60.7) | 79 (25.6) |  |  | 14 (56.0) | 13 (30.2) |  |  |
| Distal gastrectomy | 9 (32.1) | 206 (66.9) |  |  | 9 (36.0) | 27 (62.8) |  |  |
| Proximal gastrectomy | 2 (7.1) | 23 (7.5) |  |  | 2 (8.0) | 3 (7.0) |  |  |
| Surgical procedure |  |  | 0.007 |  |  |  | 0.112 |  |
| Open | 26 (92.9) | 212 (68.6) |  |  | 23 (92.0) | 33 (76.7) |  |  |
| Laparoscopic | 2 (7.1) | 96 (32.2) |  |  | 2 (8.0) | 10 (23.3) |  |  |
| T stage |  |  | <0.001 |  |  |  | 0.188 |  |
| T1 | 3 (10.7) | 195 (63.3) |  |  | 3 (12.0) | 12 (27.9) |  |  |
| T2 | 8 (28.6) | 18 (5.8) |  |  | 8 (32.0) | 6 (14.0) |  |  |
| T3 | 11 (39.3) | 49 (15.9) |  |  | 8 (32.0) | 17 (39.5) |  |  |
| T4 | 6 (21.4) | 46 (14.9) |  |  | 8 (18.6) | 8 (18.6) |  |  |
| N stage |  |  | <0.001 |  |  |  | 0.261 |  |
| N0 | 11 (39.9) | 210 (68.2) |  |  | 8 (32.0) | 16 (37.2) |  |  |
| N1 | 11 (39.3) | 26 (8.4) |  |  | 11 (44.0) | 12 (27.9) |  |  |
| N2 | 5 (17.9) | 27 (8.8) |  |  | 5 (20.0) | 7 (16.3) |  |  |
| N3 | 1 (3.6) | 45 (14.6) |  |  | 1 (4.0) | 8 (18.6) |  |  |
| M stage |  |  | 0.544 |  |  |  | 1.000 |  |
| M0 | 28 (100.0) | 304 (98.7) |  |  | 25 (100.0) | 43 (100.0) |  |  |
| M1 | 0 (0.0) | 4 (1.3) |  |  | 0 (0.0) | 0 (0.0) |  |  |
| pTNM stage |  |  | <0.001 |  |  |  | 0.913 |  |
| I | 6 (21.4) | 202 (65.6) |  |  | 6 (24.0) | 12 (27.9) |  |  |
| II | 14 (50.0) | 32 (10.4) |  |  | 11 (44.0) | 19 (44.2) |  |  |
| III | 8 (28.6) | 70 (22.7) |  |  | 8 (32.0) | 12 (27.9) |  |  |
| IV | 9 (0.0) | 4 (1.3) |  |  | 0 (0.0) | 0 (0.0) |  |  |
| **BMI**: Body Mass Index; **NEC**: neuroendocrine carcinoma; **PSM**: Propensity Score Matching | | | | | | | | |

**Supplement Table 5** Comparison of clinicopathological characteristics before and after PSM of <30%G-HMiNEN patients in neoadjuvant group

| Patient Characteristics | Unmatched comparison | | |  | Matched comparison | | |  |
| --- | --- | --- | --- | --- | --- | --- | --- | --- |
|  | <30%G-HMiNEN  (n=12) | Gastric adenocarcinoma  (n=477) | *P* value |  | <30%G-HMiNEN  (n=10) | Gastric adenocarcinoma  (n=18) | *P* value |  |
| Age (year), mean±SD | 61.7±13.3 | 58.3±10.3 | 0.267 |  | 66.0±7.7 | 66.1±7.4 | 0.970 |  |
| Gender (male/female) | 12/0 | 367/110 | 0.059 |  | 10/0 | 18/0 | 1.000 |  |
| BMI, mean±SD | 25.1±3.4 | 23.5±3.4 | 0.099 |  | 24.4±3.3 | 23.6±3.0 | 0.527 |  |
| Adjuvant therapy |  |  | 0.420 |  |  |  | 0.172 |  |
| Yes | 11 (91.7) | 459 (96.2) |  |  | 9 (90.0) | 18 (100.0) |  |  |
| No | 1 (8.3) | 18 (3.8) |  |  | 1 (10.0) | 0 (0.0) |  |  |
| Tumor location |  |  | 0.120 |  |  |  | 0.689 |  |
| Upper third | 9 (75.0) | 222 (46.5) |  |  | 8 (80.0) | 14 (77.8) |  |  |
| Middle third | 1 (8.3) | 50 (10.5) |  |  | 0 (0.0) | 2 (11.1) |  |  |
| Lower third | 1 (8.3) | 189 (39.6) |  |  | 1 (10.0) | 1 (5.6) |  |  |
| Entire | 1 (8.3) | 16 (3.4) |  |  | 1 (10.0) | 1 (5.6) |  |  |
| Tumor size |  |  | 0.033 |  |  |  | 0.638 |  |
| < 5cm | 4 (33.3) | 303 (63.5) |  |  | 3 (30.0) | 7 (38.9) |  |  |
| ≥5cm | 8 (66.7) | 174 (36.5) |  |  | 7 (70.0) | 11 (61.1) |  |  |
| Type of gastrectomy |  |  | 0.013 |  |  |  | 0.662 |  |
| Total gastrectomy | 11 (91.7) | 239 (50.1) |  |  | 9 (90.0) | 17 (94.4) |  |  |
| Distal gastrectomy | 0 (0.0) | 181 (37.9) |  |  | 0 (0.0) | 0 (0.0) |  |  |
| Proximal gastrectomy | 1 (8.3) | 57 (11.9) |  |  | 1 (10.0) | 1 (5.6) |  |  |
| Surgical procedure |  |  | 0.316 |  |  |  | 0.274 |  |
| Open | 12 (100.0) | 440 (92.2) |  |  | 10 (100.0) | 16 (88.9) |  |  |
| Laparoscopic | 0 (0.0) | 37 (7.8) |  |  | 0 (0.0) | 2 (11.1) |  |  |
| T stage |  |  | 0.545 |  |  |  | 0.523 |  |
| T1 | 0 (0.0) | 28 (5.9) |  |  | 0 (0.0) | 0 (0.0) |  |  |
| T2 | 1 (8.3) | 60 (12.6) |  |  | 1 (10.0) | 4 (22.2) |  |  |
| T3 | 6 (50.0) | 154 (32.3) |  |  | 6 (60.0) | 7 (38.9) |  |  |
| T4 | 5 (41.7) | 235 (49.6) |  |  | 3 (30.0) | 7 (38.9) |  |  |
| N stage |  |  | 0.641 |  |  |  | 0.299 |  |
| N0 | 5 (41.7) | 158 (33.1) |  |  | 5 (50.0) | 6 (33.3) |  |  |
| N1 | 1 (8.3) | 99 (20.8) |  |  | 1 (10.0) | 4 (22.2) |  |  |
| N2 | 3 (25.0) | 80 (16.8) |  |  | 3 (30.0) | 2 (11.1) |  |  |
| N3 | 3 (25.0) | 140 (29.4) |  |  | 1 (10.0) | 6 (33.3) |  |  |
| M stage |  |  | <0.001 |  |  |  | 1.000 |  |
| M0 | 10 (83.3) | 471 (98.7) |  |  | 10 (100.0) | 18 (100.0) |  |  |
| M1 | 2 (16.7) | 6 (1.3) |  |  | 0 (0.0) | 0 (0.0) |  |  |
| ypTNM stage |  |  | <0.001 |  |  |  | 0.901 |  |
| I | 1 (8.3) | 52 (10.9) |  |  | 1 (10.0) | 1 (5.6) |  |  |
| II | 5 (41.7) | 163 (34.2) |  |  | 5 (50.0) | 9 (50.0) |  |  |
| III | 4 (33.3) | 256 (53.7) |  |  | 4 (40.0) | 8 (44.4) |  |  |
| IV | 2 (16.7) | 6 (1.3) |  |  | 0 (0.0) | 0 (0.0) |  |  |
| **BMI**: Body Mass Index; **PSM**: Propensity Score Matching; **<30%G-HMiNEN**: Gastric high-grade mixed neuroendocrine-non-neuroendocrine neoplasm with neuroendocrine carcinoma components less than 30% | | | | | | | | |

**Supplement Table 6** Comparison of clinicopathological characteristics before and after PSM of G-HMiNEN patients in neoadjuvant group

| Patient Characteristics | Unmatched comparison | | |  | Matched comparison | | |  |
| --- | --- | --- | --- | --- | --- | --- | --- | --- |
|  | G-HMiNEN  (n=19) | Gastric adenocarcinoma  (n=477) | *P* value |  | G-HMiNEN  (n=18) | Gastric adenocarcinoma  (n=36) | *P* value |  |
| Age (year), mean±SD | 63.1±8.5 | 58.3±10.3 | 0.043 |  | 62.3±7.9 | 62.2±8.2 | 0.944 |  |
| Gender (male/female) | 16/3 | 367/110 | 0.459 |  | 15/3 | 27/9 | 0.487 |  |
| BMI, mean±SD | 25.0±4.0 | 23.5±3.4 | 0.060 |  | 25.0±4.1 | 23.3±3.3 | 0.123 |  |
| Adjuvant therapy |  |  | 0.388 |  |  |  | 1.000 |  |
| Yes | 19 (100.0) | 459 (96.2) |  |  | 18 (100.0) | 36 (100.0) |  |  |
| No | 0 (0.0) | 18 (3.8) |  |  | 0 (0.0) | 0 (0.0) |  |  |
| Tumor location |  |  | 0.002 |  |  |  | 0.155 |  |
| Upper third | 12 (63.2) | 222 (46.5) |  |  | 11 (61.1) | 21 (58.3) |  |  |
| Middle third | 6 (31.6) | 50 (10.5) |  |  | 6 (33.3) | 5 (13.9) |  |  |
| Lower third | 1 (5.3) | 189 (39.6) |  |  | 1 (5.6) | 8 (22.2) |  |  |
| Entire | 0 (0.0) | 16 (3.4) |  |  | 0 (0.0) | 2 (5.6) |  |  |
| Tumor size |  |  | 0.974 |  |  |  | 0.845 |  |
| < 5cm | 12 (63.2) | 303 (63.5) |  |  | 11 (61.1) | 21 (58.3) |  |  |
| ≥5cm | 7 (36.8) | 174 (36.5) |  |  | 7 (38.9) | 15 (41.7) |  |  |
| Type of gastrectomy |  |  | 0.109 |  |  |  | 0.755 |  |
| Total gastrectomy | 14 (73.7) | 239 (50.1) |  |  | 13 (72.2) | 27 (75.0) |  |  |
| Distal gastrectomy | 3 (15.8) | 181 (37.9) |  |  | 3 (16.7) | 7 (19.4) |  |  |
| Proximal gastrectomy | 2 (10.5) | 57 (11.9) |  |  | 2 (11.1) | 2 (5.6) |  |  |
| Surgical procedure |  |  | 0.689 |  |  |  | 0.610 |  |
| Open | 18 (94.7) | 440 (92.2) |  |  | 17 (94.4) | 35 (97.2) |  |  |
| Laparoscopic | 1 (5.3) | 37 (7.8) |  |  | 1 (5.6) | 1 (2.8) |  |  |
| T stage |  |  | 0.257 |  |  |  | 0.469 |  |
| T1 | 0 (0.0) | 28 (5.9) |  |  | 0 (0.0) | 0 (0.0) |  |  |
| T2 | 2 (10.5) | 60 (12.6) |  |  | 2 (11.1) | 2 (5.6) |  |  |
| T3 | 10 (52.6) | 154 (32.3) |  |  | 9 (50.0) | 14 (38.9) |  |  |
| T4 | 7 (36.8) | 235 (49.6) |  |  | 7 (38.9) | 20 (55.6) |  |  |
| N stage |  |  | 0.021 |  |  |  | 0.319 |  |
| N0 | 1 (5.3) | 158 (33.1) |  |  | 1 (5.6) | 7 (19.4) |  |  |
| N1 | 6 (31.6) | 99 (20.8) |  |  | 6 (33.3) | 8 (22.2) |  |  |
| N2 | 7 (36.8) | 80 (16.8) |  |  | 6 (33.3) | 7 (19.4) |  |  |
| N3 | 5 (26.3) | 140 (29.4) |  |  | 5 (27.8) | 14 (38.9) |  |  |
| M stage |  |  | 0.623 |  |  |  | 1.000 |  |
| M0 | 19 (100.0) | 471 (98.7) |  |  | 18 (100.0) | 36 (100.0) |  |  |
| M1 | 0 (0.0) | 6 (1.3) |  |  | 0 (0.0) | 0 (0.0) |  |  |
| ypTNM stage |  |  | 0.380 |  |  |  | 0.836 |  |
| I | 0 (0.0) | 52 (10.9) |  |  | 0 (0.0) | 0 (0.0) |  |  |
| II | 6 (31.6) | 163 (34.2) |  |  | 6 (33.3) | 11 (30,6) |  |  |
| III | 13 (68.4) | 256 (53.7) |  |  | 12 (66.7) | 25 (69.4) |  |  |
| IV | 0 (0.0) | 6 (1.3) |  |  | 0 (0.0) | 0 (0.0) |  |  |
| **BMI**: Body Mass Index; **PSM**: Propensity Score Matching; **G-HMiNEN**: Gastric high-grade mixed neuroendocrine-non-neuroendocrine neoplasm | | | | | | | | |

**Supplement Table 7** Comparison of clinicopathological characteristics before and after PSM of >70%G-HMiNEN plus pure NEC patients in neoadjuvant group

| Patient Characteristics | Unmatched comparison | | |  | Matched comparison | | |  |
| --- | --- | --- | --- | --- | --- | --- | --- | --- |
|  | >70%G-HMiNEN plus pure NEC  (n=32) | Gastric adenocarcinoma  (n=477) | *P* value |  | >70%G-HMiNEN plus pure NEC  (n=31) | Gastric adenocarcinoma  (n=62) | *P* value |  |
| Age (year), mean±SD | 59.8±8.8 | 58.3±10.3 | 0.427 |  | 60.0±8.8 | 58.6±10.2 | 0.502 |  |
| Gender (male/female) | 24/8 | 367/110 | 0.801 |  | 24/7 | 46/16 | 0.734 |  |
| BMI, mean±SD | 23.1±4.0 | 23.5±3.4 | 0.526 |  | 23.0±4.0 | 23.6±3.6 | 0.475 |  |
| Adjuvant therapy |  |  | 0.019 |  |  |  | 0.071 |  |
| Yes | 28 (87.5) | 459 (96.2) |  |  | 28 (90.3) | 61 (98.4) |  |  |
| No | 4 (12.5) | 18 (3.8) |  |  | 3 (9.7) | 1 (1.6) |  |  |
| Tumor location |  |  | 0.006 |  |  |  | 0.746 |  |
| Upper third | 25 (78.1) | 222 (46.5) |  |  | 24 (77.4) | 45 (72.6) |  |  |
| Middle third | 2 (6.3) | 50 (10.5) |  |  | 2 (6.5) | 3 (4.8) |  |  |
| Lower third | 5 (15.6) | 189 (39.6) |  |  | 5 (16.1) | 14 (22.6) |  |  |
| Entire | 0 (0.0) | 16 (3.4) |  |  | 0 (0.0) | 0 (0.0) |  |  |
| Tumor size |  |  | 0.907 |  |  |  | 0.881 |  |
| < 5cm | 20 (62.5) | 303 (63.5) |  |  | 19 (61.3) | 37 (59.7) |  |  |
| ≥5cm | 12 (37.5) | 174 (36.5) |  |  | 12 (38.7) | 25 (40.3) |  |  |
| Type of gastrectomy |  |  | 0.007 |  |  |  | 0.899 |  |
| Total gastrectomy | 25 (78.1) | 239 (50.1) |  |  | 24 (77.4) | 47 (75.8) |  |  |
| Distal gastrectomy | 4 (12.5) | 181 (37.9) |  |  | 4 (12.9) | 10 (16.1) |  |  |
| Proximal gastrectomy | 3 (9.4) | 57 (11.9) |  |  | 3 (9.7) | 5 (8.1) |  |  |
| Surgical procedure |  |  |  |  |  |  | 0.470 |  |
| Open | 30 (93.8) | 440 (92.2) |  |  | 29 (93.5) | 60 (96.8) |  |  |
| Laparoscopic | 2 (6.3) | 37 (7.8) |  |  | 2 (6.5) | 2 (3.2) |  |  |
| T stage |  |  | <0.001 |  |  |  | 0.007 |  |
| T0 | 1 (3.1) | 0 (0.0) |  |  | 0 (0.0) | 0 (0.0) |  |  |
| T1 | 3 (9.4) | 28 (5.9) |  |  | 3 (9.7) | 5 (8. 1) |  |  |
| T2 | 0 (0.0) | 60 (12.6) |  |  | 0 (0.0) | 10 (16.1) |  |  |
| T3 | 19 (59.4) | 154 (32.3) |  |  | 19 (61.3) | 18 (29.0) |  |  |
| T4 | 9 (28.1) | 235 (49.6) |  |  | 9 (29.0) | 29 (46.8) |  |  |
| N stage |  |  | 0.002 |  |  |  | 0.008 |  |
| N0 | 11 (34.4) | 158 (33.1) |  |  | 10 (32.3) | 34 (54.8) |  |  |
| N1 | 15 (46.9) | 99 (20.8) |  |  | 15 (48.4) | 11 (17.7) |  |  |
| N2 | 4 (12.5) | 80 (16.8) |  |  | 4 (12.9) | 5 (8.1) |  |  |
| N3 | 2 (6.3) | 140 (29.4) |  |  | 2 (6.5) | 12 (19.4) |  |  |
| M stage |  |  | 0.380 |  |  |  | 1.000 |  |
| M0 | 31 (96.9) | 471 (98.7) |  |  | 30 (96.8) | 60 (96.8) |  |  |
| M1 | 1 (3.1) | 6 (1.3) |  |  | 1 (3.2) | 2 (3.2) |  |  |
| ypTNM stage |  |  | <0.001 |  |  |  | 0.921 |  |
| 0 | 1 (3.1) | 0 (0.0) |  |  | 0 (0.0) | 0 (0.0) |  |  |
| I | 3 (9.4) | 52 (10.9) |  |  | 3 (9.7) | 9 (14.5) |  |  |
| II | 20 (62.5) | 163 (34.2) |  |  | 20 (64.5) | 39 (62.9) |  |  |
| III | 7 (21.9) | 256 (53.7) |  |  | 7 (22.6) | 12 (19.4) |  |  |
| IV | 1 (3.1) | 6 (1.3) |  |  | 1 (3.2) | 2 (3.2) |  |  |
| **BMI**: Body Mass Index; **NEC**: neuroendocrine carcinoma; **PSM**: Propensity Score Matching; **>70%G-HMiNEN**: Gastric high-grade mixed neuroendocrine-non-neuroendocrine neoplasm with neuroendocrine carcinoma components over 70% | | | | | | | | |

**Supplement Table 8** Comparison of clinicopathological characteristics before and after PSM of pure NEC patients in neoadjuvant group

| Patient Characteristics | Unmatched comparison | | |  | Matched comparison | | |  |
| --- | --- | --- | --- | --- | --- | --- | --- | --- |
|  | pure NEC  (n=27) | Gastric adenocarcinoma  (n=477) | *P* value |  | pure NEC  (n=26) | Gastric adenocarcinoma  (n=52) | *P* value |  |
| Age (year), mean±SD | 59.8±8.4 | 58.3±10.3 | 0.453 |  | 60.1±8.4 | 59.5±11.1 | 0.786 |  |
| Gender (male/female) | 19/8 | 367/110 | 0.433 |  | 19/7 | 38/14 | 1.000 |  |
| BMI, mean±SD | 23.3±4.2 | 23.5±3.4 | 0.818 |  | 23.2±4.3 | 23.1±3.2 | 0.874 |  |
| Adjuvant therapy |  |  | 0.006 |  |  |  | 0.191 |  |
| Yes | 23 (85.2) | 459 (96.2) |  |  | 23 (88.5) | 50 (96.2) |  |  |
| No | 4 (14.8) | 18 (3.8) |  |  | 3 (11.5) | 2 (3.8) |  |  |
| Tumor location |  |  | 0.015 |  |  |  | 0.630 |  |
| Upper third | 21 (77.8) | 222 (46.5) |  |  | 20 (76.9) | 37 (71.2) |  |  |
| Middle third | 2 (7.4) | 50 (10.5) |  |  | 2 (7.7) | 8 (15.4) |  |  |
| Lower third | 4 (14.8) | 189 (39.6) |  |  | 4 (15.4) | 7 (13.5) |  |  |
| Entire | 0 (0.0) | 16 (3.4) |  |  | 0 (0.0) | 0 (0.0) |  |  |
| Tumor size |  |  | 0.741 |  |  |  | 1.000 |  |
| < 5cm | 18 (66.7) | 303 (63.5) |  |  | 17 (65.4) | 34 (65.4) |  |  |
| ≥5cm | 9 (33.3) | 174 (36.5) |  |  | 9 (34.6) | 18 (34.9) |  |  |
| Type of gastrectomy |  |  | 0.019 |  |  |  | 0.943 |  |
| Total gastrectomy | 21 (77.8) | 239 (50.1) |  |  | 20 (76.9) | 40 (76.9) |  |  |
| Distal gastrectomy | 4 (14.8) | 181 (37.9) |  |  | 4 (15.4) | 7 (13.5) |  |  |
| Proximal gastrectomy | 2 (7.4) | 57 (11.9) |  |  | 2 (7.7) | 5 (9.6) |  |  |
| Surgical procedure |  |  | 0.947 |  |  |  | 0.212 |  |
| Open | 25 (92.6) | 440 (92.2) |  |  | 24 (92.3) | 51 (98.1) |  |  |
| Laparoscopic | 2 (7.4) | 37 (7.8) |  |  | 2 (7.7) | 1 (1.9) |  |  |
| T stage |  |  | <0.001 |  |  |  | 0.039 |  |
| T0 | 1 (3.7) | 0 (0.0) |  |  | 0 (0.0) | 0 (0.0) |  |  |
| T1 | 3 (11.1) | 28 (5.9) |  |  | 3 (11.5) | 3 (5.8) |  |  |
| T2 | 0 (0.0) | 60 (12.6) |  |  | 0 (0.0) | 10 (19.2) |  |  |
| T3 | 15 (55.6) | 154 (32.3) |  |  | 15 (57.7) | 18 (34.6) |  |  |
| T4 | 8 (29.6) | 235 (49.6) |  |  | 8 (30.8) | 21 (40.4) |  |  |
| N stage |  |  | 0.009 |  |  |  | 0.041 |  |
| N0 | 10 (37.0) | 158 (33.1) |  |  | 9 (34.6) | 28 (53.8) |  |  |
| N1 | 12 (44.4) | 99 (20.8) |  |  | 12 (46.2) | 9 (17.3) |  |  |
| N2 | 3 (11.1) | 80 (16.8) |  |  | 3 (11.5) | 5 (9.6) |  |  |
| N3 | 2 (7.4) | 140 (29.4) |  |  | 2 (7.7) | 10 (19.2) |  |  |
| M stage |  |  | 0.291 |  |  |  | 0.717 |  |
| M0 | 26 (96.3) | 471 (98.7) |  |  | 25 (96.2) | 49 (94.2) |  |  |
| M1 | 1 (3.7) | 6 (1.3) |  |  | 1 (3.8) | 3 (5.8) |  |  |
| ypTNM stage |  |  | <0.001 |  |  |  | 0.890 |  |
| 0 | 1 (3.7) | 0 (0.0) |  |  | 0 (0.0) | 0 (0.0) |  |  |
| I | 3 (11.1) | 52 (10.9) |  |  | 3 (11.5) | 9 (17.3) |  |  |
| II | 16 (59.3) | 163 (34.2) |  |  | 16 (61.5) | 29 (55.8) |  |  |
| III | 6 (22.2) | 256 (53.7) |  |  | 6 (23.1) | 11 (21.2) |  |  |
| IV | 1 (3.7) | 6 (1.3) |  |  | 1 (3.8) | 3 (5.8) |  |  |
| **BMI**: Body Mass Index; **NEC**: neuroendocrine carcinoma; **PSM**: Propensity Score Matching | | | | | | | | |
